# Development of an alternative device for measurement and characterization of selected meteorological parameters

**DOI:** 10.1038/s41598-023-35839-5

**Published:** 2023-07-07

**Authors:** A. O. Adelakun, O. Akano

**Affiliations:** Department of Physics, Federal University of Technology, Akure, Ondo Nigeria

**Keywords:** Climate sciences, Engineering, Physics

## Abstract

Weather monitoring and forecasting during some of nature’s most violent events, such as lightning and thunder, necessitates immediate preventive action for improved agricultural precision, power equipment effectiveness among others. Weather stations that are all-in-one for villages, low-income communities, and cities could provide a dependable, cost-effective, robust, and user-friendly solution. A wide range of low-cost weather monitoring stations equipped with ground-based and satellite-based lightning detectors are available on the market. This paper develops a low-cost real-time data logger device that measures lightning strikes and other weather parameters. Temperature and relative humidity are detected and recorded by the sensor (BME280). The sensing unit, readout circuit unit, microcontroller unit, recording unit, real-time clock, display unit, and power supply unit are the seven sections of the lightning detector with a real-time data logger. The sensing unit of the instrument is made of a lightning sensor glued to a polyvinyl chloride (PVC) to prevent moisture inflow and short circuit. The readout circuit consists of a 16-bit analog-to-digital converter and a filter designed to improve the output signal of the lightning detector. It was programmed with C-language and tested using the integrated development environment on the Arduino-Uno microcontroller (IDE). The device was calibrated, and its accuracy was determined using data from a standard lightning detector instrument from the Nigerian Meteorological Agency (NIMET).

## Introduction

Weather forcasting in the last decade required reliable data from modern automatic weather stations. Data generated from portable, low-cost, low-maintenance weather stations, among other requirements, is critical for modeling applications and decision-making in areas such as agricultural precision, structural damage, and power equipment damage in high-income and developed cities^1,2^. In previous studies, various sensor hardware and microcontroller-based software such as Arduino^3–5^ and Raspberry Pi devices^6,7^ were deployed and widely available in markets. Mini-stations that simultaneously record severe lightning and thunderstorms, along with other weather conditions like temperature and relative humidity, are still under development.

In light of recent findings, the National Oceanic and Atmospheric Administration (NOAA) receives thousands of claims each year from insurance companies for catastrophes caused by lightning storms, forest fires from forest administration, and plane crashes caused by high frequency during the storm (https://www.ncdc.noaa.gov/billions/events (visited on 28 September 2019)^9^). Lightning is an electrostatic discharge that occurs between electrically charged cloud regions and the earth. Lightning occurs during electrical storms and is caused by the accumulation of electrical charges within the cloud and on the earth below. Lightning is still one of the most lethal natural phenomena, killing hundreds of people each year around the world^8^. The national annual number of human fatalities and injuries provides morbid statistical information; it reflects a persistent, often under-reported, societal hazard, the scale of which is unknown^10–12^.

Despite more than a century of research on the physics and phenomenology of lightning, some processes still require more in-depth investigation^13,14^. Lightning is complicated, and it is usually accompanied by severe weather, such as hail, high wind gusts, and heavy rain^15,16^. The event is characterized by a massive electrostatic discharge caused by the circulation of warm, moist air through an unbalanced electric field in the atmosphere, accompanied by a loud thunderclap. Lightning strikes 40–50 times per second, resulting in nearly 1.4 billion flashes per year^17^. A typical cloud to ground lightning strike can be over 5 km long^18^ while a typical thunderstorm may have three or more strikes per minute at its peak^19^. Attempts to understand the phenomena (being most spectacular in nature but destructive), has been a great challenge and forms one of the well-researched area.

Despite massive research efforts, the question of “how likely is it that lightning will strike an object and cause damage?” remains unanswered. Deterministic responses are not yet possible^20^. To address these issues, it is necessary to develop mobile real-time lightning detection systems capable of detecting, measuring, and recording lightning strikes as well as some weather parameters from 1 to 45 km away. A variety of sensors will be used to detect lightning strikes and measure weather parameters such as temperature [$^{\circ }$C], humidity $[\%]$, and altitude (m).

These sensors will be linked to an Arduino Uno microcontroller, and an LCD display will be used to show lightning strikes and weather parameters. This device can be used in mines to detect the potential danger of excessive lightning strikes in order to save the lives of miners and athletes (Sports lovers). Devices such as lightning detectors and weather parameter systems can help predict atmospheric changes and their effects on the environment^21^.

The following sections of this paper are organized as follows: the first section provides an introduction to the state of the art, focusing on the problem and existing approaches. The following section is a “Material and methodology” section that describes how the project was carried out. Following that, the tests and validation performed to detect and locate lightning on a stormy day and some weather parameters are described. Finally, a conclusion section is provided to explain and justify the work’s accomplishments.

## Material and methodology

**Figure 1:**
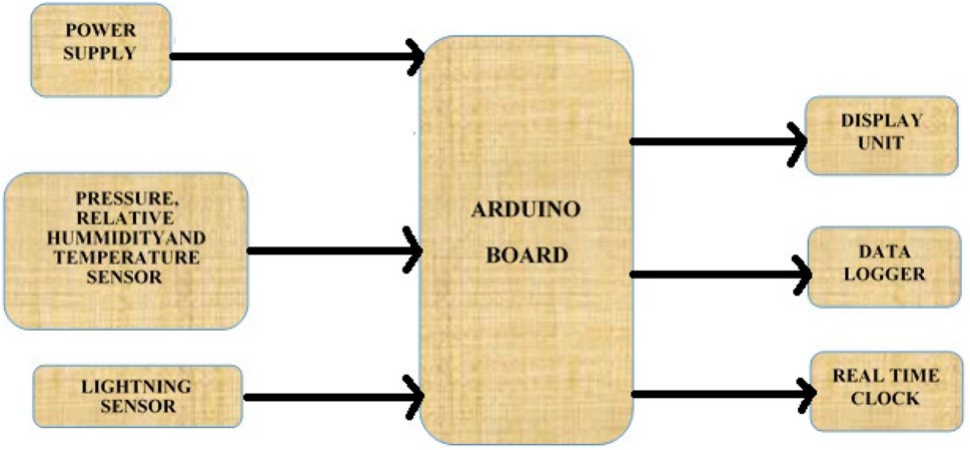
Block diagram of the designed low-cost real time data logger for lightning and weather parameters.

**Figure 2:**
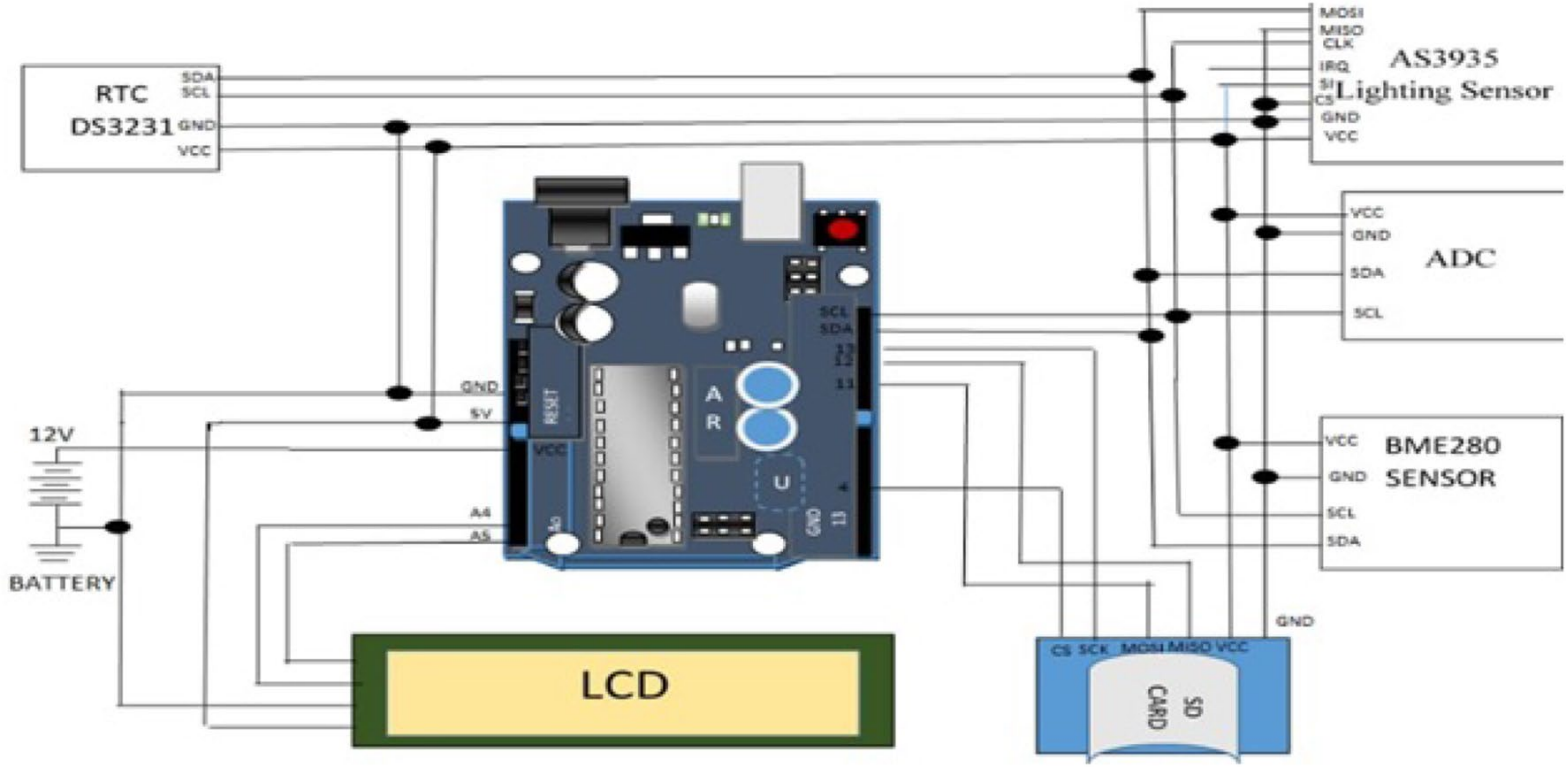
Schematic diagram of the designed low-cow real time data logger for lightning and weather parameters.

The hardware units, which include the lightning sensor AS3935, the weather sensor BME280, a 16-bit ADC converter, a real-time clock DS3231, a data logger, and an LCD, as shown in Fig. 1, and the software are used to create a low-cost real-time data logger for lightning strikes and weather parameters (the Arduino integrated development environment). By looking for lightning at a frequency of 500 kHz, an analog electrical spark produces a lightning signal pattern. An ADC will receive the analogue electrical spark and transform it into an equivalent digital electrical output. After that, a microcontroller will receive it to process. The capacitive changes that occur when the deposited material on the substrate absorbs the water molecule are what will be used as the basis for the relative humidity sensor. This capacitance value will then be calibrated against the water molecule in the atmosphere. The water molecule is eliminated when the atmosphere is dry, which causes the capacitance value to rise once more. The temperature will be measured with attitude using a semiconductor sensor. The sensor has a relative accuracy of $0.2\,^{\circ }$C. Through the microcontroller’s ADC, the temperature and relative humidity of the environment where the lightning sparks are detected are also sent to the microcontroller for processing and analogue-to-digital conversion. The C-programming language will be used to process the lightning signal that represents the temperature and relative humidity of the detected environment. The microcontroller will convert the proportional output signals into processed data using the two sensors’ proportional output signals, which send a proportional analogue signal to it. Following processing, the data would be shown on the LCD (display unit) and saved in the memory shield for later use. The lightning detector ought to be weather-resistant and capable of lasting a long time. The block diagram is shown in Fig. 1, while Fig. 2 depicts the schematic diagram for the developed real time data logger device.

## Description of the lightening main unit

The main unit of the lightning detector and weather parameters are embedded sensors unit, intelligent control unit, signal conditioning unit, display unit, data logging unit, power unit.

### Embedded sensor unit

The BOSCH Sensortec BME280 sensor measures temperature from $-40$ to $+85\,^{\circ }$C, relative humidity from 0 to 100%, and air pressure from 300 to 1100 hPa. Based on the measurement of air pressure, the height at which the measurement is taken can also be calculated. The supply voltage of this sensor spans from 1.71 to 3.6 V and it boots up in less than 2 ms. The humidity sensor’s 16-bit ADC output resolution is fixed. The AS3935 (lightning sensor) is similar in that it detects lightning and informs of lightning storm activity within a 45-km radius. The anticipated distance to the storm’s center has been reduced to 1 km. It can detect both cloud-to-ground and intra-cloud (cloud-to-cloud) flashes and includes a human-designed disturber rejection mechanism. Its customizable detection levels allow you to set thresholds for optimal control, and it works with both interconnected circuit (I2C) and serial peripheral interface (SPI) technology. Its interface is used for control as well as register reading. With a center frequency of 500 kHz and a bandwidth of 33 kHz, the AS3935 utilises narrow-band reception methods. As a result, the antenna circuit must be constructed with a resonance frequency to be more sensitive around this number.

### Intelligent control unit

Arduino is a free and open-source microcontroller that can be programmed, erased, and reprogrammed at any moment. It is made of of a physical programmable circuit board (known as a microcontroller) and a piece of software known as an IDE (Integrated Development Environment) that runs on a computer system and is used to create interactive projects (Leo, 2016). The key advantage of the Arduino UNO board over other Arduino boards is its low pricing. Bluetooth, internet, motor control, and other features are included in the device. This board has the lowest pricing when compared to other Arduino items. This is the primary reason why novices prefer this board over others. The initial aim of prototyping and quick connection of the UNOs is critical. The Arduino UNO is a low-cost, versatile, and simple-to-use programmable open-source microcontroller board that can be integrated into a wide range of electronic applications. As an output, this board can operate relays, LEDs, servos, and motors and can be interfaced with other Arduino boards, Arduino shields, and Raspberry Pi boards. The board contains digital input/output pins, analog inputs, a 16 MHz ceramic resonator, a USB connection, a power jack, an ICSP header, and a reset button. The Arduino Uno microcontroller can be powered by a DC power jack, a USB connector, or the board’s VIN pin. The team decided to power the board via the VIN pin with a 9 V battery because it is rated for voltages ranging from 7 to 12 V.

### Signal conditioning unit

The DS3231 RTC is based on the clock chip, which is driven by a temperature compensated 32 kHz crystal oscillator. The temperature compensated crystal oscillator (TCXO) offers a steady and precise reference clock, allowing the RTC to maintain an accuracy of $\pm 2$ min per year. The RTC is much more reliable now that it has a battery. SDA and SCL are linked to the Arduino Mega’s SDA and SCL pins, VCC to 5 V, and GND to the ground pin. The RTClib for Sparkfun RTC modules with DS3231 chips is used for communication between the sensor and microcontroller.

### Display unit and storage

The LCD display breakout board contains 20 communication pins labeled GND, VCC, V0, RS, R/W, E, DB0, DB1, DB2, DB3, DB4, DB5, DB6, DB7, PSB, NC, RST, VOUT, BLA, and BLK. The essential connectivity is provided by the SPI interface (SPI stands for Serial Peripheral Interface). GND was attached to the Arduino’s GND, 5–5 V, V0 to the wiper of a 10 k$\omega$ potentiometer (to adjust the contrast of the LCD’s characters or images against the background), RS to pin 10, R/W to pin 11, and E to pin 13. Only the DB0 through DB7 pins of the breakout board must be connected if parallel mode is to be considered.

The BLA is connected to the Arduino’s 5 V pin via a $220\omega$ resistor in series to lower voltage, and BLK is connected to GND. Enable pin (E) supports CLK (clock), R/W supports MOSI, and RS supports chip select (CS) (the latter must be connected to GND-pin set continuously LOW to inform the display’s controller chip that it should work in serial mode (PSB HIGH means parallel mode). SPI is not hardware mapped, which means that it does not require specific Arduino pins. However, the Arduino pins required to link CLK, MOSI, and CS must be defined in the display constructor. Different pins can be used as long as they are properly stated in the display constructor. A MicroSD card was also linked to the Arduino mega 2560 board. The Arduino mega 2560 was attached to the micro SD card’s clock pin, chip select pin, MOSI pin, and MISO pin.

The digital Secure Card Arduino library was activated to assist with the storage of the temperature, relative humidity, and data on lightning strikes. The temperature of the Arduino-UNOs’ environment, however, has a significant impact on their lifespan. According to a general rule, each $+10\,^{\circ }$C halves their lives. They can therefore live anywhere from 2 to 3 years (in a hot environment) and 10 to 15 years (in a cold environment), depending on the ambient temperature. The temperature throughout the research was between +19 and $+36\,^{\circ }$C. Additionally, the most frequent issues associated with UNOs are (1) choosing the wrong port for uploading the code, (2) syntax and declaration errors in Arduino code, and (3) missing libraries. In the same vein, damages occur when excessive current is drawn from the board, pins are shorted, EEPROAM wear, and when overvoltage is applied to power.

## Test and caliberation

Testing for the lightning sensor was carried out using a piezo-electric effect igniter. After the AS3935 was connected to the microcontroller and the piezo electric igniter was pressed. The spring loaded hammer hit the quartz in order to create a spark. The number of strike, distance, date and time of the event was shown on the LCD. As the piezo electric ignition is continuously pressed, the number of strikes kept increasing. The air hydrator was used to blow air against the humidity sensor and the humidity value increase and for the temperature, heat was brought near it and the temperature value increases. All the above processes indicate that the components are responding appropriately. Temperature sensor BME 280 was compared with a mercury-in-glass thermometer. By comparing BME 280 sensor with a mercury-in-glass thermometer, the following apparatus was used: a copper calorimeter, a thermometer with a temperature range of $-10$ to 120$\,^{\circ }$C, a small stove, and water in a small container. Water is poured inside the copper calorimeter, and the tip of the BME 280 temperature sensor and thermometer were inserted into the calorimeter via a non-conducting led, including the stirrer for stirring the water uniformly for even distribution of temperature. The calorimeter was placed on the hot stove, and the temperature was taken every 10 min until the water boiled. A humidity sensing section from BME 280 was developed with an Arduino Uno for test measurement. This is to determine the accuracy of the humidity section. The RH device was installed within a Steven screen at the Federal University of Technology meteorological garden in Akure that contained dry and wet thermometers. The displayed value of the developed device and the readings from dry and wet thermometers were recorded every 10 min for 2 h. When it began to rain, the lightning device was activated to take its reading. For calibration, the data from the developed lightning device was compared to data from the Nigerian Meteorological Agency (NiMET). The lightning data collected from NiMet and the data gained from the developed lightning device, along with the lightning distance observed.

## Results and discussion

**Figure 3:**
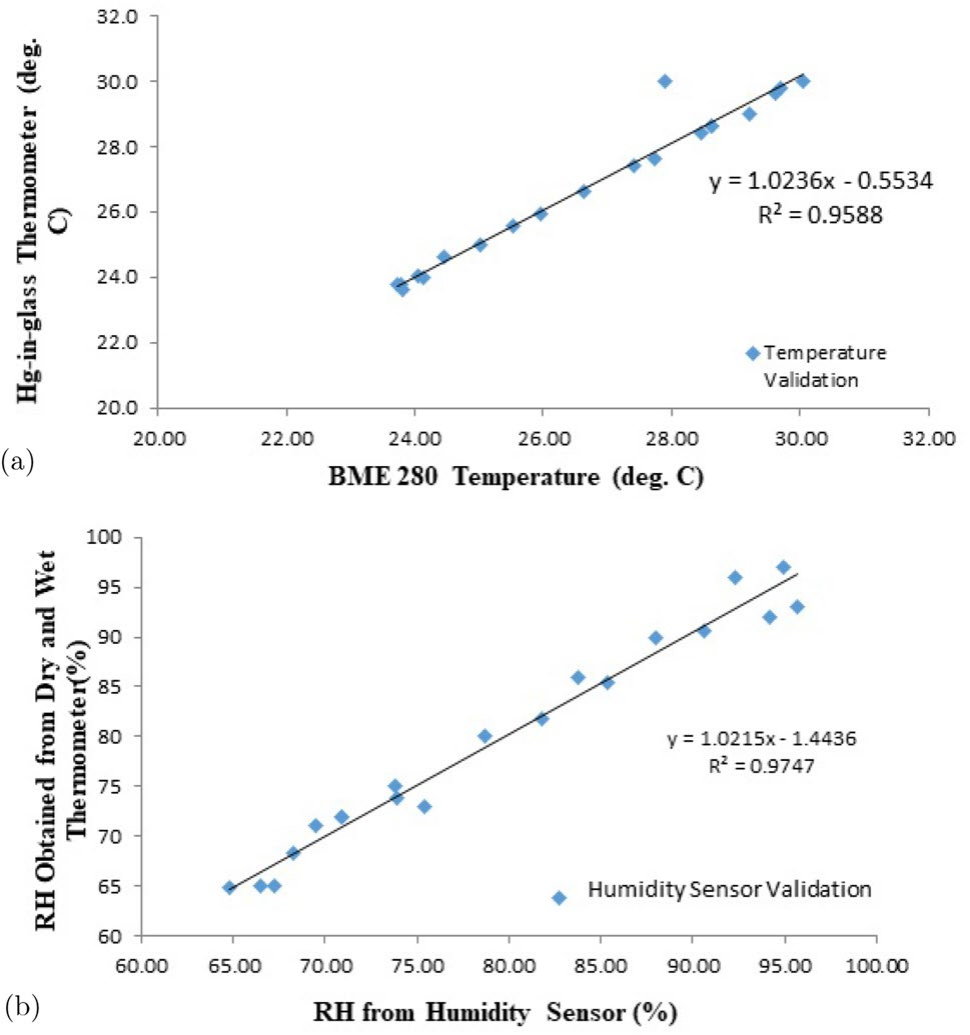
Comparing performance of BME 280 with thermometer (**a**) temperature and (**b**) relative humidity.

On the newly developed lightning instrument, performance tests and examinations were carried out. The instrument was discovered to detect the presence and potentially hazardous lightning activity in the area, as well as provide an estimate of the distance to the storm’s head. The response time was less than 10 s, indicating that the developed instrument had a fast logging time. The temperature sensor device measures temperature with a resolution of $$0.25\,^{\circ }$$C between $$- 40 $$ and $$85 \,^{\circ }$$C with $$1\%$$ accuracy, humidity with a maximum range of roughly $$99\%$$ with $$3\%$$ accuracy. Table 1 shows the accuracy of the temperature sensor (BME 280) and the Relative Humidity measurement, while the statistic analysis of the temperature sensor compared with a mercury-in-glass thermometer data were captured in Fig. 3a,b, respectively.

**Table 1:** Examination of the accuracy of the temperature sensor (BME 280) and the relative humidity measurement.

Time (10 min)	Temperature		Relative humidity	
	T_BME$^o{C}$	T_Hg$^o{C}$	RH_BME%	RH_Hg (wet and dry)%
10	27.40	27.4	75.41	73.00
20	27.74	27.6	73.84	75.00
30	29.63	29.6	65.00	65.00
40	29.69	29.8	65.00	65.00
50	30.06	30.0	64.77	65.00
60	28.47	28.4	69.50	71.00
70	29.21	29.0	68.26	68.00
80	28.62	28.6	72.00	72.00
90	28.62	28.6	70.90	72.00
100	27.90	30.0	73.91	74.00
110	26.65	26.6	80.00	80.00
120	25.96	26.0	82.00	82.00

**Figure 4:**
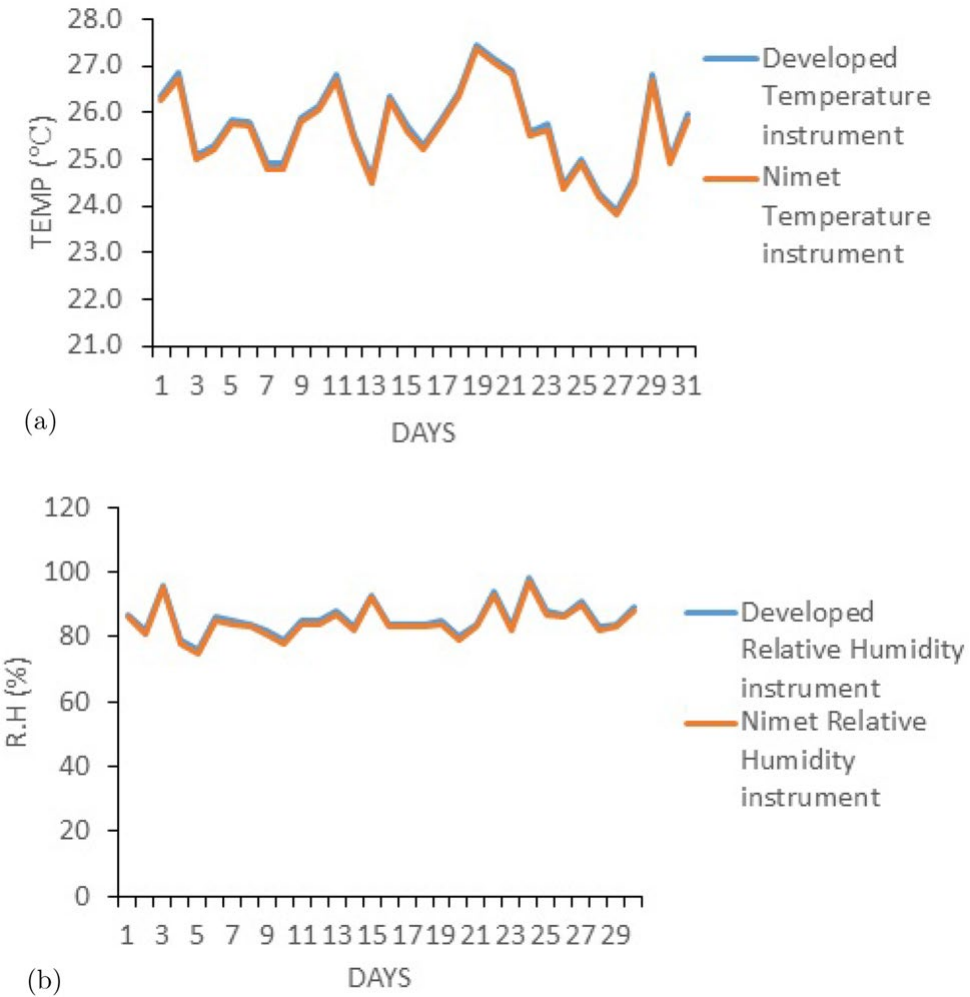
Comparism graph for (**a**) temperature sensor and (**b**) relative humidity sensor.

**Figure 5:**
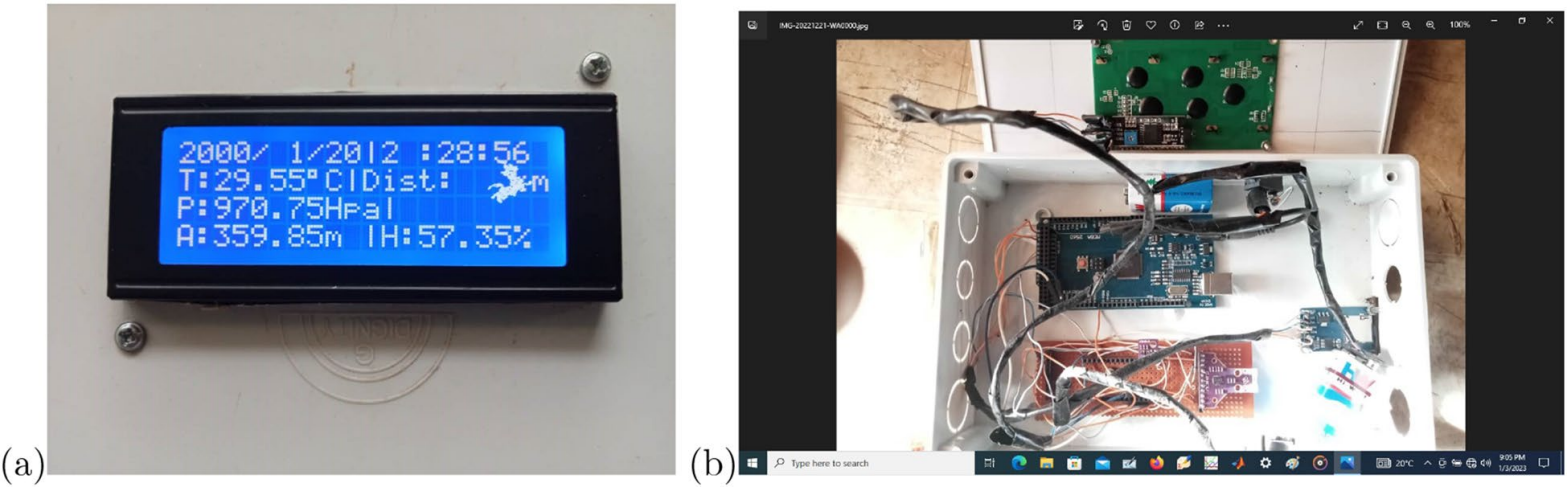
Physical realization of the proposed device (**a**) front phase and (**b**) hardware construction inside casing.

In order to track down lightning strikes during the rainy season and evaluate the accuracy of certain atmospheric weather parameters, the developed lightning measuring instrument was deployed in a few different locations. The correlation between the data from the developed device and the Standard meteorological device is shown in Fig. 4a,b. The developed measurement system is represented by the blue line on the correlation graph, while the compared measurement system is represented by the red line. The close overlap of the two lines in the correlation demonstrates how closely the two instruments are related. Temperature and relative humidity are estimated to have correlations of 0.9409 and 0.9871, respectively, for two systems. Figure 5a,b shows the physical realization of the proposed device.

Table 2 displays the observations made from an open location in Kubwa (Abuja) throughout the entire month of July. Lightning, temperature, humidity, the date, and distance were all displayed on the LCD at regular intervals. In order to find lightning strikes during the rainy season and to check the accuracy of some atmospheric weather parameters, the developed lightning measuring instrument was taken to other locations. Measurements were conducted in Dutse (Abuja) in August. In the month of September, additional measurements were made in Gwarinpa (Abuja).

**Table 2:** Data acquired when comparing the developed instrument and NiMet lightning measurement.

Days	Caliberation data		Other measurements	
	NiMet device	Kubwa environment	Dutse environment	Gwarinpa environment
1		OFF	OFF	OFF
2		OFF	ON	OFF
3	Yes	ON	OFF	OFF
4		OFF	OFF	OFF
5		OFF	OFF	ON
6	Yes	ON	OFF	OFF
7		OFF	ON	OFF
8	Yes	ON	ON	OFF
9	Yes	ON	OFF	ON
10	Yes	ON	OFF	ON
11		OFF	OFF	ON
12		OFF	OFF	ON
13		OFF	ON	ON
14		OFF	OFF	OFF
15	Yes	ON	ON	ON
16		OFF	ON	ON
17		OFF	OFF	OFF
19		OFF	ON	ON
20	Yes	ON	OFF	ON
21	Yes	ON	OFF	ON
22		OFF	OFF	OFF
23		OFF	ON	OFF
24		OFF	OFF	ON
25	Yes	ON	OFF	OFF
26		OFF	OFF	OFF
27	Yes	ON	OFF	ON
28	Yes	ON	OFF	ON
29		OFF	ON	ON
30		OFF	OFF	OFF
31		OFF	OFF	OFF

A lower number of lightning occurrences were recorded in August compared to September, according to data collected at Dutse (Abuja). This observation is in line with the annual precipitation, which in the central region ranges from 1000 to 1500 mm (40–60 in.). The average minimum and maximum temperatures for August were $19.6\,^{\circ }$C and $32.2\,^{\circ }$C, respectively. The average daily rainfall for the month of August was 265 mm. The sunshine hours decrease for a longer period of time in the central region, where the rainy season lasts longer, with an average of 5 h per month. From the measurements made at Gwarinpa (Abuja), it was found that September had a higher number of lightning occurrences than July and August. This observation is consistent with the central region’s annual precipitation, which is between 40 and 60 in. or 1000–1500 mm. The average minimum and maximum temperatures for September were $19.0\,^{\circ }$C and $32.6\,^{\circ }$C, respectively. In September, there is typically 255 mm of precipitation over 16 days. In the central region, where the rainy season lasts a longer time, September sees a longer decline in sunshine hours than July and August—an average of 6 h.

## Concluding remarks

The performance of the lightning detector with meteorological parameters indicated good response, when compared to available lightning data received from a conventional device. The intra-cloud (cloud-to-cloud) and cloud-to-ground lightning measuring ranges are 1–45 km. Aside from the fact that the device is water resistant and long-lasting, the processing data will be preserved in the memory shield for future use. The lightning detector technology is simple to service, maintain, and repair. The lightning detector is water-resistant and capable of long-lasting. The system’s performance review demonstrates that its continuous measurement can be utilized for lightning monitoring to examine how lightning impacts human health, communication signals, the environment, and energy distribution (transmission cable) over time.

## Data Availability

Data sharing is not applicable to this article as no new data were created or analyzed in this study.
